# Long non‐coding RNA EWSAT1 promoted metastasis and actin cytoskeleton changes via miR‐24‐3p sponging in osteosarcoma

**DOI:** 10.1111/jcmm.16121

**Published:** 2020-11-22

**Authors:** Dewei Shen, Yize Liu, Yuexin Liu, Tao Wang, Lin Yuan, Xuyang Huang, Yong Wang

**Affiliations:** ^1^ 4^th^ Department of Orthopaedic Surgery Central Hospital affiliated to Shenyang Medical College Shenyang China; ^2^ School of Basic Medical Sciences Shenyang Medical College Shenyang China; ^3^ 2^nd^ Department of Orthopaedic Surgery Second Affiliated Hospital of Shenyang Medical College Shenyang China; ^4^ 2^nd^ Department of Neurology Central Hospital affiliated to Shenyang Medical College Shenyang China; ^5^ Central Laboratory Central Hospital Affiliated to Shenyang Medical College Shenyang China

**Keywords:** actin stress fibre formation, lncRNAEWSAT1, metastasis, miR‐24‐3p, osteosarcoma

## Abstract

Non‐coding RNAs are closely associated with tumorigenesis in multiple malignant tumours, including osteosarcoma (OS). Long non‐coding RNA Ewing sarcoma‐associated transcript 1 (EWSAT1) plays a role in metastasis, and actin cytoskeletal changes in OS remain unclear. In the current study, we showed that EWSAT1 expression was up‐regulated in OS and that an elevation in the EWSAT1 expression level was correlated with poor prognosis in patients with OS. Functionally, we showed that knockdown of EWSAT1 suppressed migration and induced actin stress fibre degradation in MNNG/HOS and 143B cells. Moreover, we found that ROCK1 was a key downstream effector in EWSAT1‐mediated cell migration and actin stress fibre changes. Furthermore, we demonstrated that ROCK1 and EWSAT1 shared a similar microRNA response element of microRNA‐24‐3p (miR‐24‐3p). Moreover, we verified that miR‐24‐3p suppressed ROCK1 and its mediated migration and actin stress fibres change by direct targeting. EWSAT1 promoted ROCK1‐mediated migration and actin stress fibre formation through miR‐24‐3p sponging. Lastly, through an in vivo study, we demonstrated that EWSAT1 promoted lung metastasis in OS. According to the above‐mentioned results, we suggest that EWSAT1 acts as an oncogene and that EWSAT1/miR‐24‐3p/ROCK1 axial could be a new target in the treatment of OS.

## 1 INTRODUCTION

As the most prevalent primary sarcoma in young adolescence, osteosarcoma (OS) commonly develops at the metaphysis of long bones and produces a bone or osteoid.^1^ The highest aggressive phenotypes and fast growth and early stage of pulmonary metastasis are the main factors for the unfavourable prognosis of OS.^2^ According to an epidemiological study, approximately 20% of patients had pulmonary metastasis in their first clinical visit.^1^ Although a combined treatment, involving tumorectomy and chemotherapy, significantly improves the survival rate of patients with OS, the survival period of the involved individuals who present with lung metastasis remains non‐optimistic.^3^, ^4^ Therefore, there is an urgent need for determining available metastatic molecules and identifying their underlying mechanisms in OS.

Long non‐coding RNAs (lncRNAs), with a length of >200 nucleotides, belong to a class of non‐protein‐coding transcriptions and are extensively involved in a crowd of cell biological events. LncRNAs may function as scaffolds or guide in regulating interactions between proteins and genes and can also act as enhancers to modulate transcription of their targets.^5^, ^6^, ^7^ EWSAT1, also named LINC00277, was first reported as an oncogenic transcription in Ewing sarcoma.^8^ Presently, related studies on EWSAT1 are rare. Through a protein array analysis and RNA immunoprecipitation assay, Marques reported that EWSAT1 regulated gene expression partially through interaction with the heterogeneous nuclear ribonucleoprotein.^8^ Sun reported that EWSAT1 positively regulated lncRNA MEG3 expression at the transcriptional level and that EWSAT1 promoted OS cell growth and metastasis.^9^ It is well known that lncRNAs can also function by acting through microRNA (miRNA) sponging or competitive endogenous RNA (ceRNA), which was first proposed by Leonardo Salmena in 2011.^10^ The CeRNA hypothesis is that all types of RNA transcripts communicate through a new ‘‘language’’ mediated by miRNA‐binding sites (‘‘microRNA response elements,’’ or ‘‘MREs’’).^10^ Our previous studies revealed that lncRNAs, including taurine‐up‐regulated gene 1 (TUG1), metastasis‐associated lung adenocarcinoma transcript 1 (MALAT1), differentiation antagonizing non‐protein coding RNA (DANCR) and nuclear‐enriched abundant transcript 1 (NEAT1) exerted their oncogenic functions in OS, colorectal cancer and ovarian cancer through the ceRNA mechanism.^11^, ^12^, ^13^, ^14^, ^15^ Here, we considered EWSAT1, a new lncRNA, as a research point and illustrated the oncogenic role of EWSAT in OS, especially in metastasis and actin stress fibre formation.

## 2 MATERIALS AND METHODS

### 2.1 Patients and tissue samples

We collected 50 OS tissue specimens and paired paratumour tissue specimens during tumorectomy at Liaoning Cancer Hospital & Institute between January 2015 and January 2018. All cases were diagnosed as OS according to the clear histologic diagnosis of OS and staged according to the TNM classification of the International Union Against Cancer. Written informed consent was provided by the patients whose tissues were used in this study. The Institute Research Medical Ethics Committee of Central Hospital Affiliated to Shenyang Medical College and Institute Research Medical Ethics Committee of Liaoning Cancer Hospital & Institute approved this study.

### 2.2 Cell culture

The human osteoblast cell line hFOB 1.19 was cultured in Dulbecco's modified Eagle's medium (DMEM)/F12 (Gibco). The human OS cell lines MG‐63, U2OS, MNNG/HOS and 143B were cultured in DMEM (Gibco). HEK‐293 cells were cultured in MEM (Gibco). All culture media were supplemented with 10% (v/v) foetal bovine serum (FBS; Sigma), 100 IU/mL penicillin (Baomanbio) and 100 mg/mL streptomycin (Baomanbio). All OS cell lines and HEK‐293 were incubated at 37°C, while hFOB 1.19 was cultured at 34°C in a humidified atmosphere containing 5% CO_2_. The cultured cells were passaged when they grew to 80% confluence.

### 2.3 Reverse transcription and quantitative real‐time PCR

The procedure was conducted as previously described.^13^ Total RNAs were isolated using TRIzol reagent (Invitrogen). A Takara RNA PCR kit (Takara) was applied to synthesize cDNA according to the manufacturer's protocol. PCR assays containing SYBR Premix Ex Taq II (Takara) were performed according to the manufacturer's manual. U6 small nuclear RNA and GAPDH were used as internal controls. Primer sequences, as presented in Table 1, were synthesized by RiboBio Co., Ltd.

**Table 1:** Primer sequences used in the present research

Gene	Sequences of primers used
EWSAT1‐F for PCR	AGAAAGGGCTGTGACAGCAT
EWSAT1‐R for PCR	TCCCTCCTTCCACCTTCC
ROCK1‐F for PCR	AGGAAGGCGGACATATTAGTCCCT
ROCK1‐R for PCR	AGACGATAGTTGGGTC CCGGC
GAPDH‐F for PCR	GCACCGTCAAGGCTGAGAAC
GAPDH‐R for PCR	TGGTGAAGACGCCAGTGGA
miR‐24‐3p‐F for PCR	GGGTGGCTCAGTTCAGCAG
miR‐24‐3p‐R for PCR	CAGTGCGTGTCGTGGAGT
miR‐335‐5p‐F for PCR	GGGTCAAGAGCAATAACGAA
miR‐335‐5p‐R for PCR	CAGTGCGTGTCGTGGAGT
miR‐144‐3p‐F for PCR	GGGTACAGTATAGATGA
miR‐144‐3p‐R for PCR	CAGTGCGTGTCGTGGAGT
U6‐F for PCR	CTCGCTTCGGCAGCACA
U6‐R for PCR	AACGCTTCACGAATTTGCGT
EWSAT1‐01 siRNA	GCACAGCATCCTTGCTCTA
EWSAT1‐02 siRNA	GGAGTTATCTGGGTATCAA
miR‐24‐3p mimics	TGGCTCAGTTCAGCAGGAACAG
miR‐24‐3p inhibitor	CTGTTCCTGCTGAACTGAGCCA
ISH Probe for EWSAT1	CTGAGCCCAGGTATATATCTAACAGAAG
ISH Probe for miR‐24‐3p	GGCTCAGTTCAGCAGGAA

### 2.4 Oligonucleotide and plasmid transfection

Effective siRNA oligonucleotides that targeted EWSAT1 (1# si‐EWSAT1 and 2# si‐EWSAT1) and the corresponding control siRNA (si‐con), miR‐24‐3p oligonucleotides including miR‐24‐3p mimics and mimic control, miR‐24‐3p inhibitors and inhibitor control were synthesized by RiboBio. Oligonucleotide sequences are also shown in Table 1. EWSAT1 overexpression plasmids (oe‐EWSAT1) and ROCK1 overexpression plasmids (oe‐ROCK1) were chemically synthetized by GenePharma. When MNNG/HOS and 143B cells reached 70‐80% confluence, the aforementioned oligonucleotides and plasmids were transfected to the cultured OS cells using Lipofectamine 3000 (Invitrogen) according to the manufacturer's instructions. Y‐27632 dihydrochloride (Abcam) was used as a selective ROCK1 blocker.

### 2.5 In situ hybridization (ISH) assay

The procedure of ISH and IHC was performed as previously described.^16^ Briefly, specific probes targeting EWSAT1 and miR‐24‐3p were purchased from Boster Bio Co., Ltd. The probes were added to the hybridization solution and hybridized according to the manufacturer's protocol. After an incubation of slides with 4‐nitroblue‐tetrazolium for 30 minutes at 25°C and with nuclear fast red for 5 minutes at room temperature, the slices were observed and photographed under a microscope (Leica).

For the IHC assay, OS tissue specimens were fixed in 10% FBS at room temperature for 1 day and embedded in paraffin. Then, the embedded tissues were treated in order: paraffin‐embedding, 4 μm thickness of the section, deparaffinization, rehydration, hydrogen peroxide incubation, antigen retrieval, 10% goat serum (BioWorld) blocking, first antibody incubation (Anti‐ROCK1, Abcam) at 4°C overnight, secondary antibody incubation (Goat Anti‐Rabbit IgG H&L, Abcam) at 37°C for 20 minutes, streptavidin‐horseradish peroxidase complex incubation, diaminobenzidine tetrahydrochloride (MedChemExpress) stain, haematoxylin (Amresco) and counterstain. All sections were assessed by two experienced pathologists individually.

### 2.6 Transwell assays

The procedure was conducted as previously described.^14^ Briefly, OS cells were seeded on upper chambers (BD Bioscience). Culture medium with and without 10% FBS was supplemented into the lower and upper wells, respectively, and the entire set‐up was incubated for 24 hours. On the subsequent day, non‐migrated cells were wiped out. Then, the filters were fixed in 90% ethanol, followed by crystal violet staining. Five random fields were counted per chamber using an inverted microscope (Olympus).

### 2.7 Rhodamine phalloidin immunofluorescence

The actin filaments in OS cells were stained with rhodamine phalloidin as previously reported.^17^ Briefly, OS cells that reached 70‐80% confluence were fixed in 4% formaldehyde for 10 minutes at room temperature and permeabilized with 0.3% Triton X‐100 in PBS for 15 minutes. Then, the cells were incubated with 1 × rhodamine phalloidin (Abcam) working solution at room temperature for 30 minutes and observed under a fluorescent microscope (Leica, Wetzlar, Germany). Images were analysed using Image‐Pro Plus 6.0 software (Media Cybernetics).

### 2.8 Western blot analysis

The procedure was conducted as previously described.^14^ Briefly, proteins were extracted with radioimmunoprecipitation assay lysis buffer (Sigma) and electrophoretically transferred onto PVDF membranes (Amresco). The membranes were incubated first with special primary antibodies that, respectively, probed ROCK1 (anti‐ROCK1, Abcam, dilution rates of 1:2000), lysophosphatidic acid acyltransferase β (anti‐LPAATβ, Abcam, concentration of 1 µg/mL), and tyrosine kinase non‐receptor 2 (anti‐TNK2, Abcam, dilution rates of 1:50) at 4°C overnight and then with secondary antibodies (Abcam, dilution rates of 1:2000) at 25°C for 1 hour on the following day. Protein bands were detected on an X‐ray film using an enhanced chemiluminescence detection system.

### 2.9 Dual‐luciferase reporter assay

Wild and mutant reporter plasmids that contain wild or mutant miR‐24‐3p binding sites, EWSAT1‐wt and EWSAT1‐mut and ROCK1‐wt and ROCK1‐mut, were synthesized by GenePharma. The procedure was performed as previously described.^18^ Briefly, when HEK293 cells achieved 70% confluence, EWSAT1‐wt or ROCK1‐wt and EWSAT1‐mut or ROCK1‐mut were co‐transfected with miR‐24‐3p mimics and mimic control using Lipofectamine 3000 (Invitrogen), respectively. After 48 hours, luminescence changes in each group were determined using a Dual‐Luciferase Reporter Assay System (Promega) according to the manufacturer's protocol.

### 2.10 RNA pull‐down assay

The procedure was conducted as previously described.^19^ LncRNA‐EWSAT1‐wt and lncRNA‐EWSAT1‐wt were transcribed from vector pGEM^®^‐T (Promega) and biotin‐labelled with the Biotin RNA Labeling Mix (Roche, Basel, Switzerland) and T7 RNA polymerase (Roche), treated with RNase‐free DNase I (Roche), and purified using an RNeasy Mini Kit (Qiagen). The biotinylated EWSAT1 probes were dissolved in binding and washing buffer and incubated with Dynabeads M‐280 Streptavidin (Invitrogen) at 25°C for 10 minutes to generate probe‐coated beads according to the manufacturer's protocol. Then, cell lysates of MNNG/HOS and 143B were incubated with probe‐coated beads, and the RNA complexes bound to these beads were eluted and extracted for qRT‐PCR analysis to detect the relative expression of miRNAs.

### 2.11 Xenograft nude mouse model

Six‐week‐old female BALB/c nude mice were purchased from the Animal Care and Use Committee of Dalian Medical University Ltd. and maintained under sterile‐specific pathogen‐free conditions. Moreover, 200 μL PBS containing 1 × 10^6^ MNNG/HOS cells with stable overexpressed EWSAT1 or control vector (pMSCV) were intravenously injected into nude mice (n = 6 per group) for the evaluation of lung metastasis. The lungs of each group were harvested for further detection after 6 weeks. This study was performed in accordance with the Guide for the Care and Use of Laboratory Animals of the National Institutes of Health and approved by the Institute Research Medical Ethics Committee of Central Hospital Affiliated to Shenyang Medical College. All efforts were made to minimize animal suffering, reduce the number of animals used and utilize possible alternatives to in vivo techniques.

### 2.12 Statistical analysis

All experiments were repeated in triplicate, and all data from three independent experiments were expressed as mean ± SD. GraphPad Prism version 5.0 (GraphPad Software, Inc) software and SPSS 19.0 statistical software were used for conducting statistical analysis. Pearson's chi‐square test or Fisher's exact test was used to analyse the correlation between EWSAT1 and clinicopathological features of patients with OS; furthermore, log‐rank test was used for survival analysis using GraphPad Prism version 5.0. Differences in the two groups were analysed using Student's *t* test or one‐way analysis of variance. Differences were considered significant if *P* < .05.

## 3 RESULTS

### 3.1 EWSAT1 was elevated and correlated with poor prognosis in patients with OS

**Figure 1:**
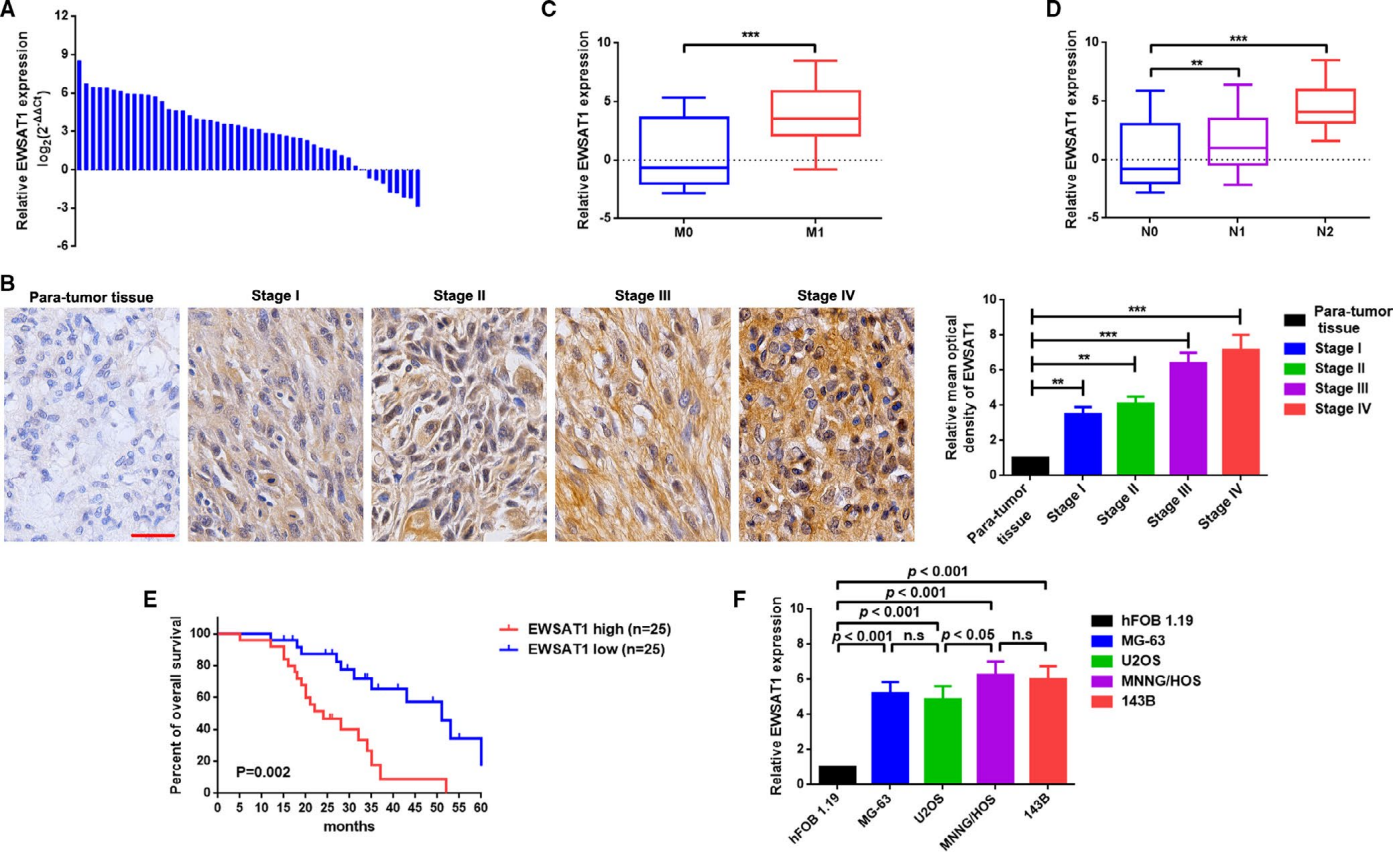
Elevated EWSAT1 was correlated with poor prognosis in patients with osteosarcoma (OS). A, Expression of EWSAT1 in OS tissue specimens was determined by qRT‐PCR assay; data are shown as ^△^ *C*t. B, EWSAT1 was significantly elevated in patients with advanced staging as determined by in situ hybridization assay. **P < .001 when normalized and compared with the paratumour tissue group. C,D, EWSAT1 expression was remarkably elevated in patients with distant metastasis (C) and lymph node metastasis (D). **P < .01, **P < .001, when normalized and compared with the M_0_ or N_0_ group, respectively. E, Kaplan‐Meier analyses indicated that the overall survival of patients with high EWSAT1 expression was significantly shorter than that of patients with low EWSAT1 expression, *P* = .002. F, EWSAT1 expression was elevated in the OS cell lines MG‐63, U2OS, MNNG/HOS and 143B as determined by the qRT‐PCR assay. *P‐*values are shown in the diagram, and n.s. means non‐significant. Data are shown as mean ± SD from three independent experiments

EWSAT1 expression in the collected OS tissue specimens was determined using qRT‐PCR. As presented in Figure 1A, EWSAT1 had high expression in most (43/50, 86.00%) OS tissue specimens compared with that of paratumour tissue specimens. Additionally, ISH was applied to measure the expression of EWSAT1 in different stages of OS tissue specimens. As shown in Figure 1B, the expression of EWSAT1 gradually increased with advanced staging (*P* < .001). Further, we found that elevated EWSAT1 expression was more commonly present in OS tissue specimens with distant metastasis and lymph node metastasis (Figure 1C,D, *P* < .001). Furthermore, we analysed the correlation between EWSAT1 expression and clinicopathological features of patients with OS. As shown in Figure 1E and Table 1, patients with OS were divided into the high‐ and low‐EWSAT1 groups according to the expression using a median method. Moreover, high EWSAT1 expression was closely correlated with shorter survival rate (Figure 1E *, P = *.002), advanced staging (IIB/III) (*P = *.008) and distant metastasis (*P = *.016) (Table 2). Eventually, EWSAT1 expression in the normal human osteoblastic cell line hFOB 1.19 and the four OS cell lines MG‐63, U2OS, HOS and 143B was determined using qRT‐PCR. As shown in Figure 1F, EWSAT1 expression was significantly up‐regulated in four OS cell lines compared with that in hFOB 1.19 (*P* < .001).

**Table 2:** Association of EWSAT1 expression with clinicopathological features of osteosarcoma

Features	No. of cases	EWSAT1		*P*‐value^a^
		High	Low	
Age at diagnosis				
<18	31	18	13	.605
≥18	19	14	5	
Gender				
Female	28	17	11	.768
Male	22	15	7	
Histological subtype				
Osteoblastic	10	6	4	.896
Chondroblastic	11	8	3	
Fibroblastic	12	7	5	
Mixed	17	11	6	
Clinical stage				
I+IIA	23	10	13	.008
IIB/III	27	22	5	
Distant metastasis				
Absent	21	9	12	.016
Present	29	23	6	
Tumour size (cm)				
<5	22	13	11	.239
≥5	28	19	7	
Anatomic location				
Tibia/femur	26	18	8	.557
Elsewhere	24	14	10	

^a^ *P*‐value obtained from Pearson chi‐square test or Fisher's exact test.

### 3.2 Knockdown of EWSAT1 suppressed migration and induced actin stress fibres dissolution in MNNG/HOS and 143B cells

**Figure 2:**
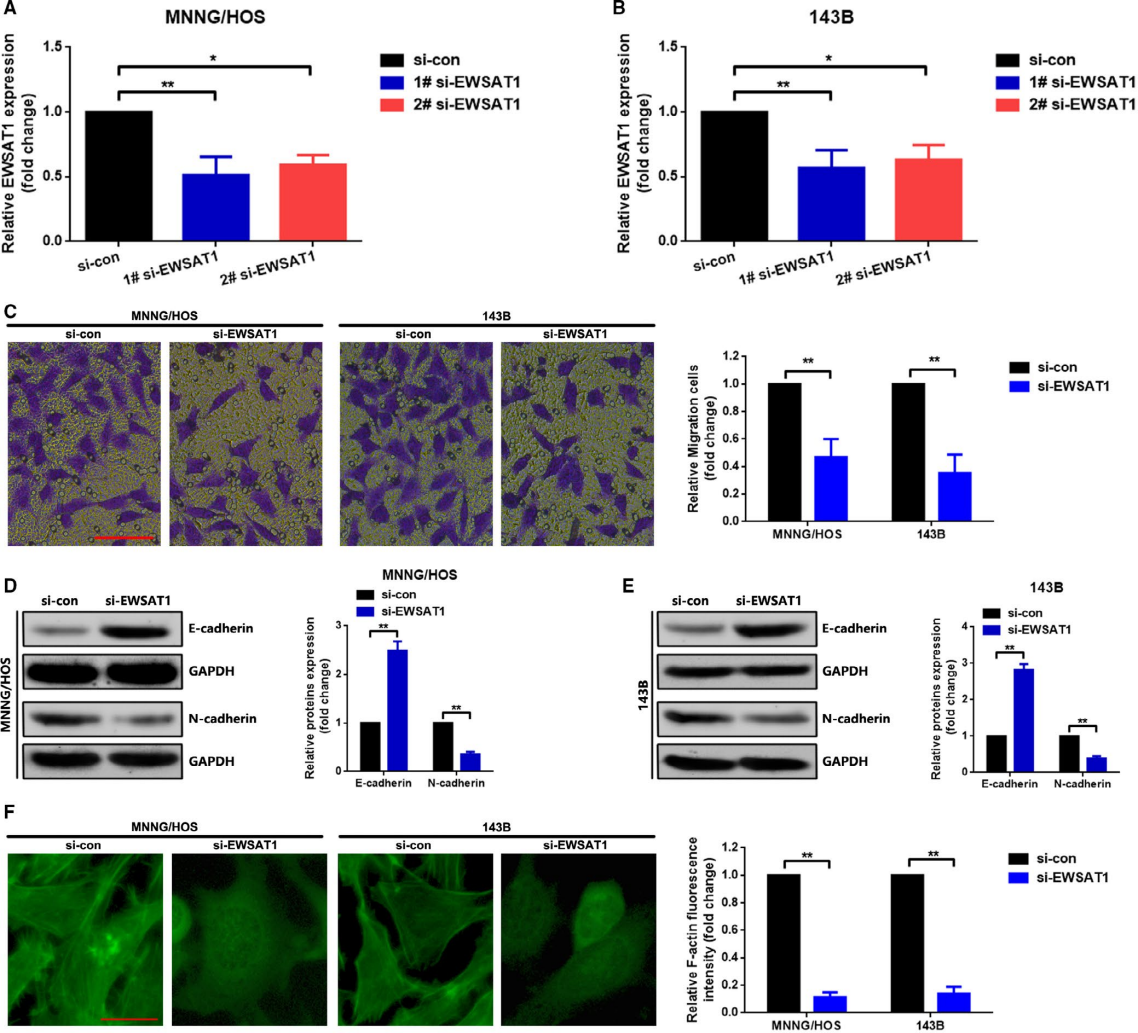
Knockdown of EWSAT1 suppressed migration and induced actin stress fibre dissolution in MNNG/HOS and 143B cells. A,B, EWSAT1 was knocked down in MNNG/HOS (A) and 143B (B) cells as confirmed by qRT‐PCR assay. C, Down‐regulation of EWSAT1 suppressed the migration of osteosarcoma (OS) cells as determined from the Transwell assay. D,E, Expression of E‐cadherin and N‐cadherin was determined by Western blot assay. F, Knockdown of EWSAT1 induced the dissolution of actin stress fibres in OS cells as determined from rhodamine phalloidin immunofluorescence. *P < .05 and **P < .01 when normalized and compared with the si‐con group, respectively. Data are shown as mean ± SD from three independent experiments

In the previous section, we elucidated that high EWSAT1 expression was closely related to distant metastasis and lymph node metastasis. Therefore, we attempted to explore the role of EWSAT1 in cell migration at the cellular level. We first knocked down the expression of EWSAT1 in MNNG/HOS and 143B cells in an RNAi experiment. As shown in Figure 2A,B, EWSAT1 was knocked down by transfection of EWSAT1 siRNA (compared with si‐con, 1# si‐EWSAT1 presented a higher silence efficacy and was selected as the silencing tool in the following RNAi experiments, *P* < .01). Second, Transwell assay was performed to evaluate the effect of EWSAT1 on OS cell migration. As presented in Figure 2C, knockdown of EWSAT1 suppressed cell migration in MNNG/HOS and 143B cells (*P* < .01). Epithelial‐to‐mesenchymal transition (EMT) is extensively reported as a key process in cancer cell metastasis.^20^ Consequently, we determined the role of EWSAT1 in OS cell EMT. As presented in Figure 2D,E, knockdown of EWSAT1 suppressed N‐cadherin but promoted E‐cadherin expression.

Reorganization of actin cytoskeleton is the initial power of cell motility and is essential for the migration of most cancer cells.^21^ Accordingly, we detected the effect of EWSAT1 on actin cytoskeleton changes. As presented in Figure 2F, knockdown of EWSAT1 also induced dissolution of actin stress fibres (*P* < .01).

### 3.3 EWSAT1 promoted migration and actin stress fibre formation by up‐regulation of ROCK1 in MNNG/HOS and 143B cells

**Figure 3:**
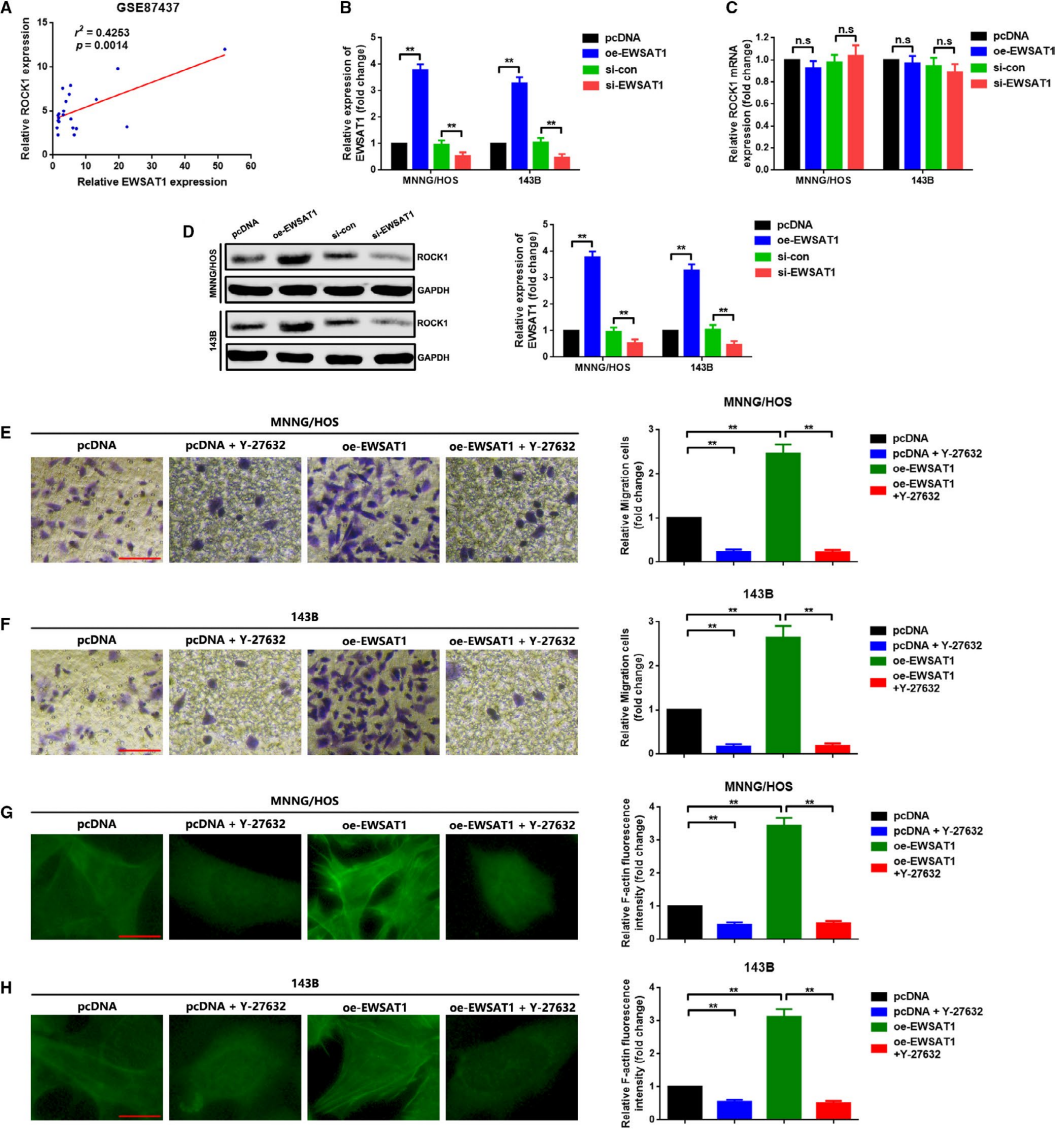
A, The relationship between ROCK1 and EWSAT1 in GEO data sets 87437 was determined by Spearman correlation analysis, *r* ^2^ = .4253, *P *= .0014. B, Expression of EWSAT1 after transfection of oeEWSAT1 and si‐EWSAT1 was confirmed using qRT‐PCR assay. **P < 0.01 when normalized with the pcDNA group and compared with the pcDNA and si‐con groups, individually. C, Up‐ and down‐regulation of EWSAT1 presented no effect on ROCK1 mRNA expression, which were confirmed using qRT‐PCR assay. **P < .01 when normalized with the pcDNA group and compared with the pcDNA and si‐con groups, individually. D, ROCK1 protein expression was positively regulated by EWSAT1 as determined using the Western blot assay. **P < .01 when normalized with the pcDNA group and compared with the pcDNA and si‐con groups, respectively. E,F, The migration ability of osteosarcoma (OS) cells was significantly enhanced by transfection of oe‐EWSAT1, while the facilitative effect was remarkably attenuated using Y‐27632 dihydrochloride—the selective ROCK1 blocker. G,H, Overexpression of EWSAT1 promoted actin stress fibre formation, but the facilitative effect was reversed using Y‐27632 dihydrochloride. **P < .01 when normalized with the pcDNA group and compared with the oe‐EWSAT1 group. Data are shown as mean ± SD from three independent experiments

Our study and other previous studies demonstrated that ROCK1 was a key molecule in migration and actin stress fibre formation in OS cells. Here, we attempted to explore whether ROCK1 was a downstream effector of EWSAT1. By analysing GEO datasets GSE87437, we first displayed that the expression of ROCK1 was positively correlated with EWSAT1 (Figure 3A). Second, we revealed that an elevation or depression of EWSAT1 positively regulated ROCK1 expression at the protein level but not at the mRNA level (Figure 3B‐D, *P* < .01). Third, Y‐27632 dihydrochloride—a selective ROCK1 inhibitor—was applied to investigate the potential role of ROCK1 played in EWSAT1 in promoting OS cell migration and actin stress fibre formation. As presented in Figure 3D,E, Y‐27632 dihydrochloride dramatically attenuated EWSAT1‐induced migration and actin stress fibre formation in MNNG/HOS and 143B cells (*P* < .01). Briefly, the outcomes of this section showed that EWSAT1 up‐regulated the expression of the ROCK1 protein to promote migration and actin stress fibre formation in MNNG/HOS and 143B cells.

### 3.4 EWSAT1 up‐regulated ROCK1 through sponging of miR‐24‐3p

**Figure 4:**
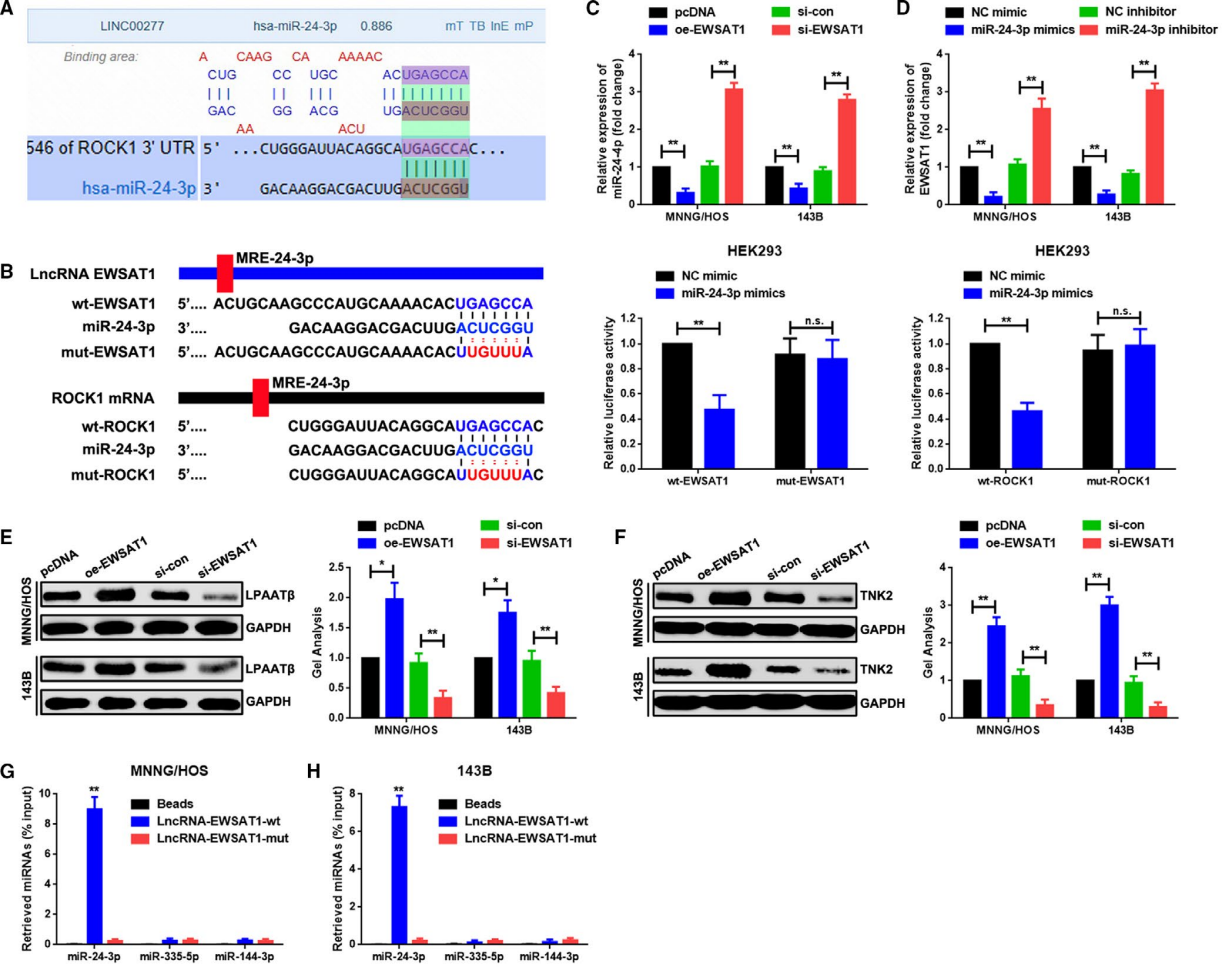
EWSAT1 up‐regulated ROCK1 through the sponging of miR‐24‐3p. A, EWSAT1 and ROCK1 shared similar MREs for miR‐24‐3p as predicted online using DIANA‐LncBase (http://carolina.imis.athena-innovation.gr) and Targetscan (http://www.targetscan.org/vert_71). B, Dural luciferase assay demonstrated that it was a cotransfection of miR‐24‐3p mimics and wt‐EWSAT1/wt‐ROCK1 rather than a cotransfection of miR‐24‐3p mimics and mut‐EWSAT1/mut‐ROCK1 that led to an evident weakening of luminescence. **P < .01, n.s*. P* > .05 when normalized and compared with the mimic control group. C,D, EWSAT1 and miR‐24‐3p regulated each other in a reciprocal manner. **P < .01 when normalized and compared with the pcDNA and NC mimic groups, individually. E,F, Up‐ and down‐regulation of EWSAT1 positively regulated LPAATβ and TNK2 expression at the protein level as determined using Western blot assay. *P < .05, **P < .01 when normalized and compared with the pcDNA group. G,H, Cell lysates collected from MNNG/HOS and 143B cells were incubated with biotin‐labelled lncRNA‐EWSAT1‐wt and lncRNA‐EWSAT1‐mut individually. It was lncRNA‐EWSAT1‐wt rather than lncRNA‐EWSAT1‐mut that could pull down miR‐24‐3p. However, neither miR‐335‐5p nor miR‐144‐3p was pulled down by any of the two plasmids (lncRNA‐EWSAT1‐wt and lncRNA‐EWSAT1‐mut). **P < .01 when normalized and compared with the beads group. Data are shown as mean ± SD from three independent experiments

It is well known that lncRNAs could regulate their target genes through a mechanism involving ceRNA. We focused on whether any miRNAs might serve as a bridge between EWSAT1 and ROCK1. Through an online prediction, miR‐24‐3p was viewed for its similar MREs for both EWSAT1 and ROCK1 (Figure 4A). Then, through a luciferase assay, we verified that EWSAT1 and ROCK1 were both targeted by miR‐24‐3p through similar miR‐24‐3p response elements (MREs‐24‐3p) (Figure 4B). Further, we elucidated that EWSAT1 and miR‐24‐3p affected each other's expression in a reciprocal manner (Figure 4C,D, *P* < .01). Furthermore, we illustrated that an elevation or depression in EWSAT1 expression regulated not only ROCK1 (Figure 3A,B, *P* < .01) but also LPAATβ and TNK2^22^, ^23^—two other verified downstream targeted genes of miR‐24‐3p (Figure 4E,F, *P* < .01). Lastly, through a RNA pull‐down assay, we demonstrated that biotin‐labelled lncRNA‐EWSAT1‐wt (containing wild MREs‐24‐3p) rather than lncRNA‐EWSAT1‐mut (containing mutant MREs‐24‐3p) precipitated miR‐24‐3p, which again proved the binding effect between EWSAT1 and miR‐24‐3p. More convincingly, the results of the RNA pull‐down assay also indicated that neither lncRNA‐EWSAT1‐wt nor lncRNA‐EWSAT1‐mut pulled down miR‐335‐5p and miR‐144‐3p—two previously verified miRNAs that targeted ROCK1^14^, ^15^ (Figure 4G,H, *P* < .01). Generally, the above‐mentioned data demonstrated strongly that EWSAT1 promoted ROCK1 through sponging of miR‐24‐3p.

### 3.5 MiR‐24‐3p suppressed ROCK1‐mediated migration and actin stress fibre formation in MNNG/HOS and 143B cells

**Figure 5:**
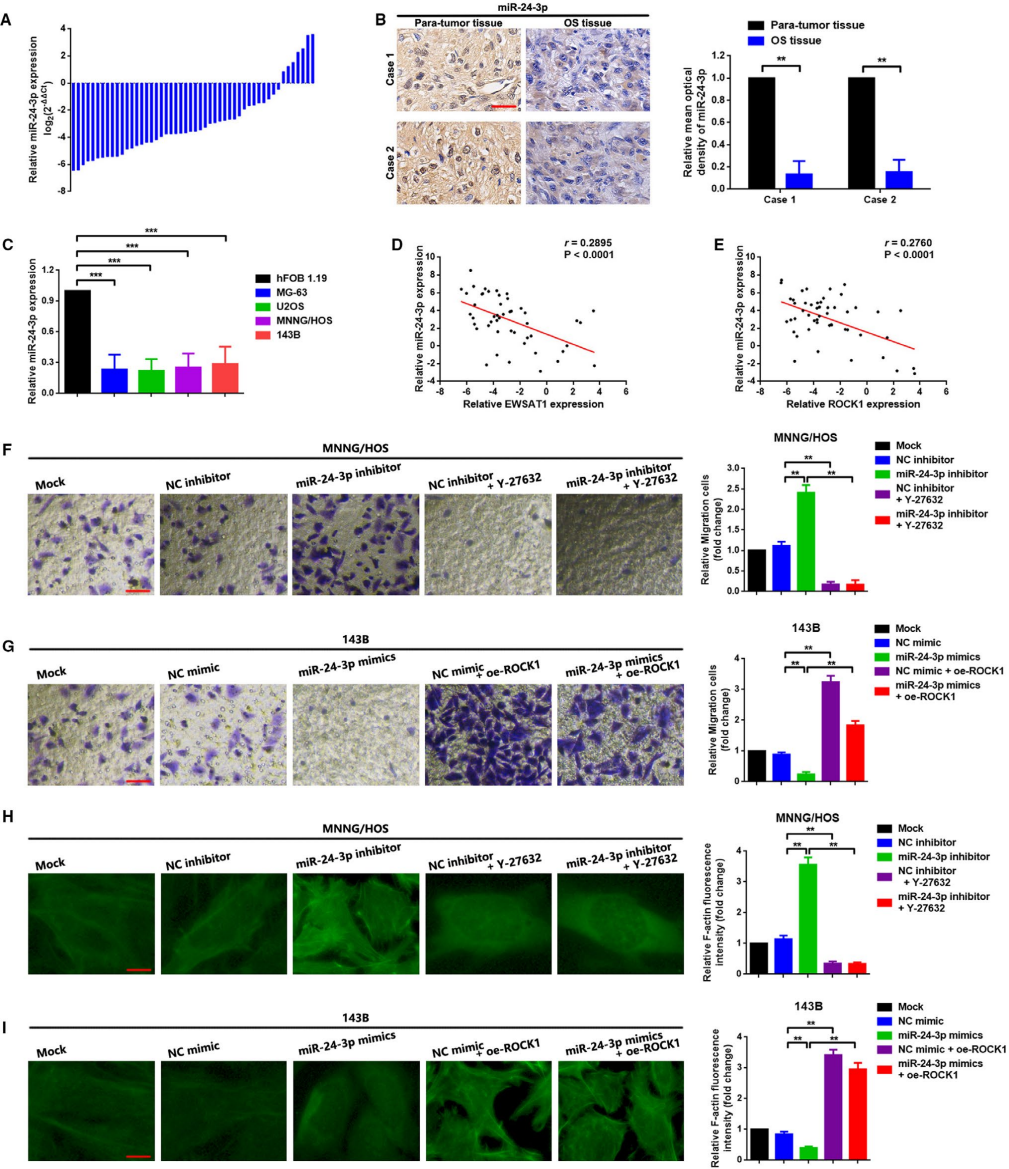
A,B, MiR‐24‐3p expression in osteosarcoma (OS) tissue specimens was measured using the qRT‐PCR assay (data are shown as △Ct) and in situ hybridization assay. **P < .01 when normalized and compared with the paratumor tissue group. C, MiR‐24‐3p expression was down‐regulated in the OS cell lines MG‐63, U2OS, MNNG/HOS and 143B as determined by the qRT‐PCR assay. ***P < .001 when normalized and compared with the hFOB1.19 group. D, A negative correlation between EWSAT1 and miR‐24‐3p was confirmed by Pearson correlation analysis, *P* < .0001. E, The same tendency was observed between ROCK1 and miR‐24‐3p as shown using Pearson correlation analysis, *P* < .0001. F,G,H,I, Knock‐down of miR‐24‐3p promoted OS cell migration and actin stress fibre formation, while the facilitative effect could be reversed using Y‐27632 dihydrochloride. On the contrary, up‐regulation of miR‐24‐3p inhibited OS cell migration and actin stress fibre formation, while the suppressive effect was reversed using the ROCK1 overexpression plasmid oe‐ROCK1. **P < .01 when normalized with the Mock group and compared with the miR‐24‐3p inhibitor group or the miR‐24‐3p mimics group, separately. Data are shown as mean ± SD from three independent experiments

In this section, we focused on the function of miR‐24‐3p working on OS cell migration and actin stress fibre formation. First, we observed that miR‐24‐3p was down‐regulated in OS tissue specimens and cell lines (Figure 5A‐C). Second, we elucidated negative correlations between miR‐24‐3p and ROCK1, miR‐24‐3p, and EWSAT1 (Figure 5D,E, *P* < .0001). Lastly, we demonstrated that depression in miR‐24‐3p expression promoted migration and actin stress fibre formation, while the facilitative effect could be reversed by Y‐27632 dihydrochloride (Figure 5F,G, *P* < .01). Thus, combined with the targeted binding effect between miR‐24‐3p and ROCK1 3′‐UTR (Figure 4B), the outcomes of this section indicated that miR‐24‐3p was an upstream regulator of ROCK1 and its mediated migration and actin stress fibre formation.

### 3.6 EWSAT1 promoted lung metastasis of OS in vivo

**Figure 6:**
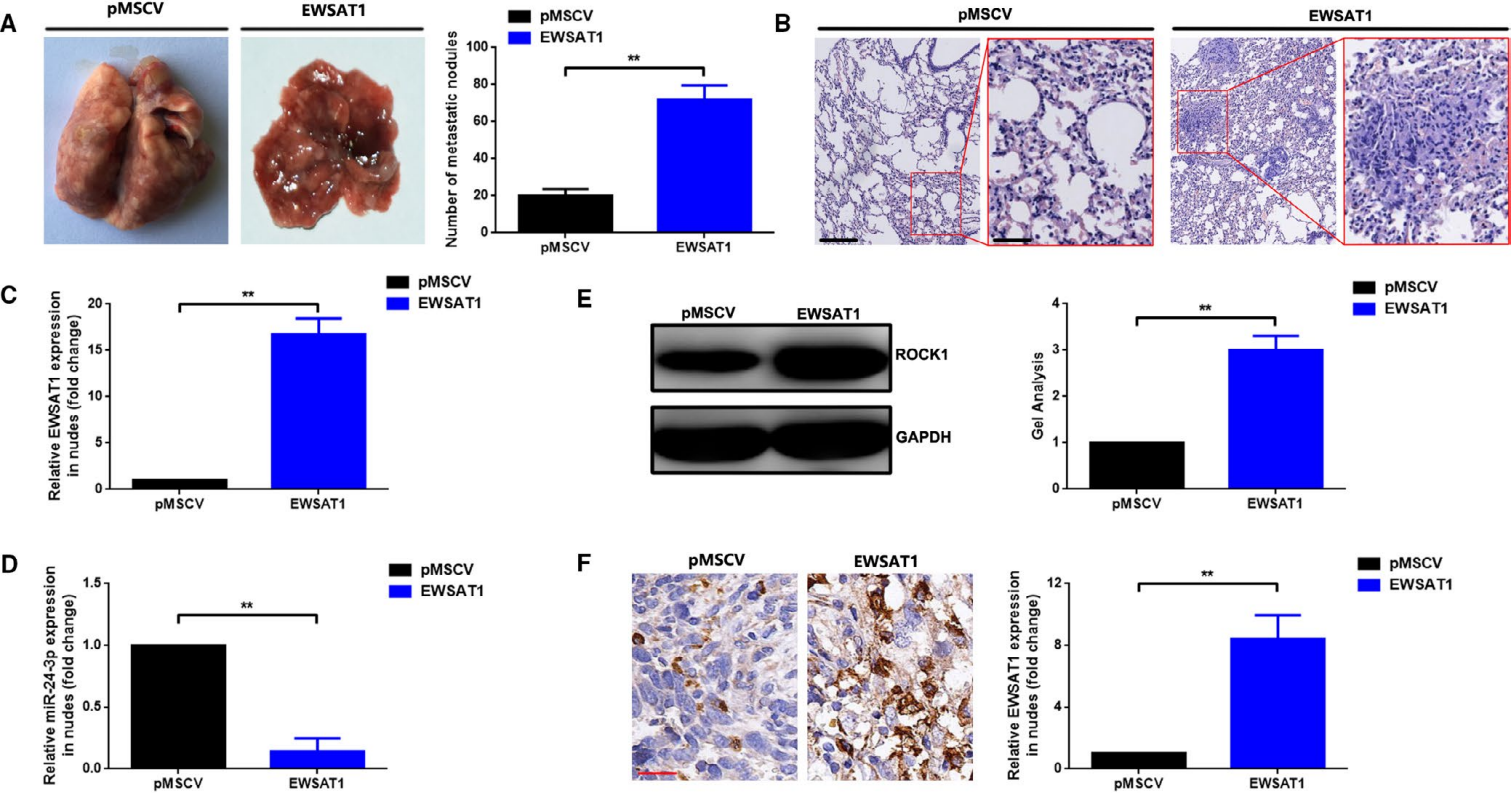
EWSAT1 promoted lung metastasis of osteosarcoma (OS) in vivo. A, Representative photos of the metastatic nodules in the lungs. B, Overexpression of EWSAT1 promoted lung metastasis of OS as presented by the representative haematoxylin‐eosin (HE) staining (scale bar of 200 μm for a magnification of 100× and scale bar of 50 μm for a magnification of 400×, respectively). C, EWSAT1 expression in the metastatic nodules was qualified using the qRT‐PCR assay. D, MiR‐24‐3p expression in the metastatic nodules was also measured using the qRT‐PCR assay. E,F, Expression of the ROCK1 protein in the metastatic nodules was determined using the Western blot assay (E) and IHC assay (F), respectively. **P < .01 when normalized and compared with the pMSCV group. Data are shown as mean ± SD from three independent experiments

In this section, xenograft mouse models were constructed to verify the function of EWSAT1 in OS in vivo. MNNG/HOS cells with stably overexpressing EWSAT1 and with corresponding vector were inoculated intravenously to the mice. As shown in Figure 6A,B (*P* < .01), the number of microscopic metastatic tumour nodules in the lungs in the EWSAT1 group was significantly higher than that in the pMSCV group. We further detected the expression of EWSAT1, miR‐24‐3p and ROCK1 in each group. As presented in Figure 6C‐F, up‐regulated EWSAT1 and ROCK1 expression but down‐regulated miR‐24‐3p expression were found in the EWSAT1 group compared with those in the pMSCV group (*P* < .01).

## 4 DISCUSSION

Increasing evidence suggests that non‐coding RNAs, including lncRNAs, miRNAs and circular RNAs (circRNAs), comprehensively participate in diverse diseases and cellular processes.^24^, ^25^, ^26^ EWSAT1, which contains four exons, is located at human chromosome 15q23 and reported as an oncogene in several cancers including ovarian cancer, nasopharyngeal carcinoma, Ewing sarcoma and OS.^8^, ^9^, ^27^, ^28^, ^29^ In the present study, we observed that EWSAT1 expression was up‐regulated in the OS tissue and that a higher EWSAT1 was correlated with shorter survival rate (*P* = .002), higher advanced TNM staging (*P* = .008) and rapid distant metastasis (*P* = .016). Moreover, through loss‐of‐function experiments, we verified that EWSAT1 promoted migration and actin stress fibre formation in two OS cell lines: MNNG/HOS and 143B. Furthermore, through an in vivo animal study, we demonstrated that overexpression of EWSAT1 promoted lung metastasis of OS. Therefore, we thought that EWSAT1 would act as a tumour initiator in OS, especially in OS metastasis.

Cancer cell metastasis is a multistage process involving invasion into the surrounding tissue, intravasation, transit into the blood or lymph, extravasation and growth at a new site.^30^ Cell migration, which has a fundamental role in tumour invasion and metastasis, is a very complicated issue, and reorganization of the actin cytoskeleton produces the necessary force for cell migration.^21^ The actin cytoskeleton plays a critical role in tumour cell migration and movement.^31^ Cai reported that ectopic expression of miR‐23a promoted dissolution of actin stress fibres in PC‐3 and DU145 cells.^32^ It is well uncovered that some classical signalling pathways, including ras homolog family (Rho) and ROCK signalling pathway, play a vital regulative effect in cytoskeletal reorganization.^33^, ^34^, ^35^ Cai found that knockdown of lncRNA MALAT1 reduced the number of actin stress fibres and suppressed metastasis through the down‐regulation of ROCK1 in the OS cell.^17^ In our previous studies, we also demonstrated that ROCK1 was a key molecule in OS cell metastasis.^12^, ^13^, ^15^, ^36^, ^37^ In the present study, we illustrated that blockage of ROCK1 significantly impaired EWSAT1‐induced migration and cytoskeletal changes, and this phenomenon indicated that ROCK1 was a substrate of EWSAT1 in OS cells.

miRNAs are another type of non‐coding RNAs that are 22‐25 nt in length. MiRNAs play critical roles in various cell biological behaviours, including proliferation, apoptosis, cell cycle control, cell differentiation and metastasis.^38^, ^39^, ^40^ As a member of the miRNA family, miR‐24‐3p (also known as miR‐24) is extensively involved in oncogenesis and progression of multiple cancers, including OS. Liu found that miR‐24‐3p significantly suppressed OS cell metastasis mediated by inhibiting Ack1 expression.^22^ Through in vitro and in vivo studies, Song reported that miR‐24‐3p inhibited OS cell proliferation by targeting LPAATβ.^23^ In the present study, we also found that miR‐24‐3p was down‐regulated and served as a bridge between EWSAT1 and ROCK1 in OS. Through luciferase assay and RNA pull‐down assay, we illustrated that miR‐24‐3p regulated EWSAT1 and ROCK1 expression by direct targeting. Functionally, miR‐24‐3p is involved in ROCK1‐mediated metastasis and cytoskeletal changes. Recently, a prevalent theory on lncRNA, miRNA and mRNA is the ceRNA theory. The CeRNA hypothesis is that all types of RNA transcripts could undergo cross‐talk through a new ‘‘language’’ mediated by similar MREs. Through a series of qRT‐PCRs, we clarified the reciprocal effect between EWSAT1 and miR‐24‐3p. Further, we showed that EWSAT1 positively regulated ROCK1, LPAATβ and TNK2, three downstream targets of miR‐24‐3p. Moreover, through the RNA pull‐down assay, we demonstrated that lncRNA‐EWSAT1‐wt pulled down only miR‐24‐3p but not miR‐335‐5p and miR‐144‐3p—two previously verified miRNAs that targeted ROCK1. These results strongly demonstrated that EWSAT1 served as a ceRNA of ROCK1 through sponging of miR‐24‐3p.

## 5 CONCLUSION

**Figure 7:**
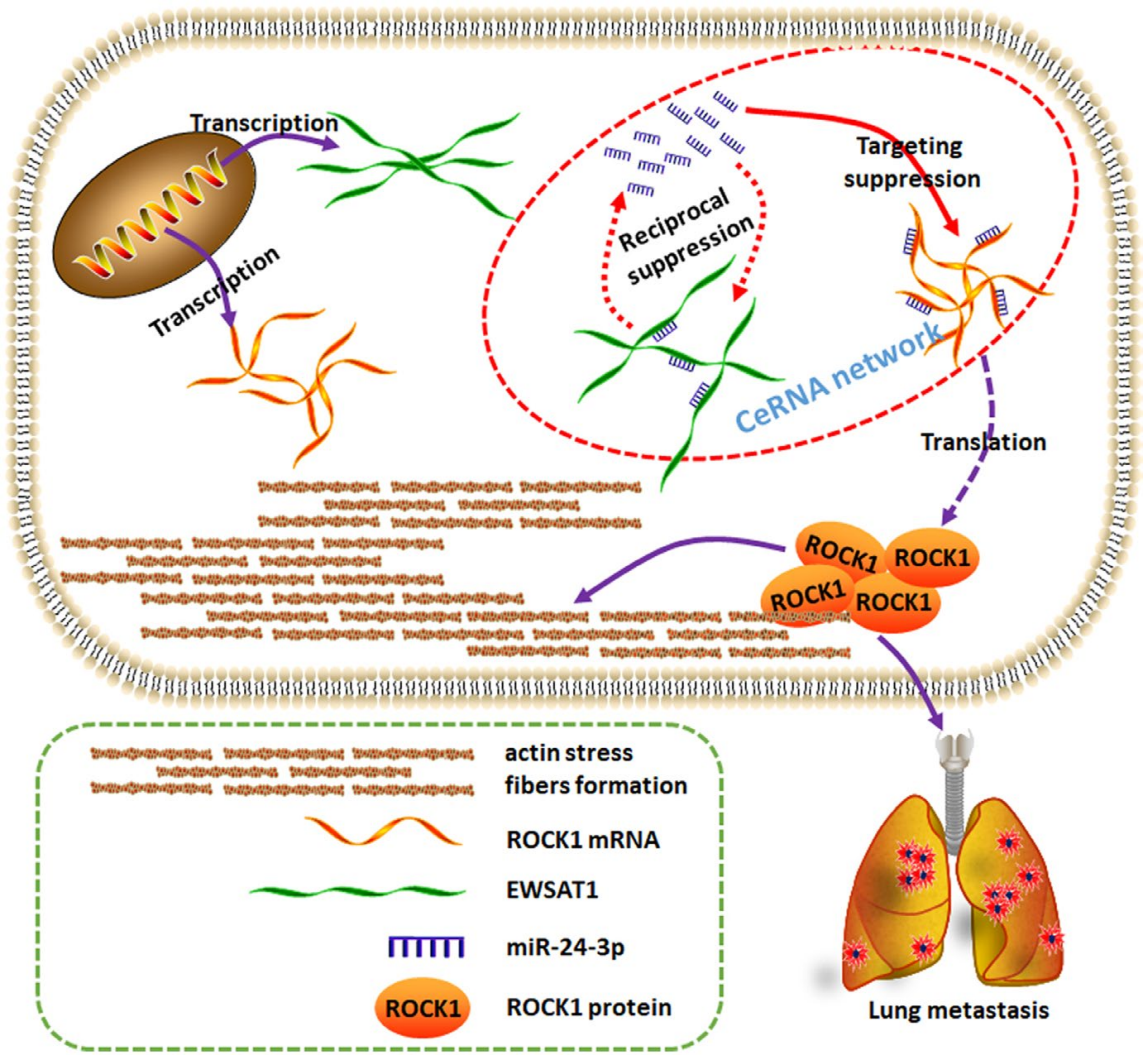
Schematic diagram of the mechanism. LncRNA EWSAT1 promoted metastasis and actin cytoskeleton changes through miR‐24‐3p sponging in osteosarcoma (OS)

Generally, as presented in Figure 7, all results of our study illustrated that EWSAT1 regulated ROCK1 and mediated migration and actin cytoskeletal changes through miR‐24‐3p decoying. Our present study proposed a new targeted axial in the treatment of OS.

## CONFLICT OF INTEREST

The authors declare no conflicts of interest.

## AUTHOR CONTRIBUTION

**Dewei Shen:** Investigation (equal). **Yize Liu:** Investigation (equal). **Yuexin Liu:** Investigation (equal). **Tao Wang:** Investigation (supporting). **Lin Yuan:** Investigation (supporting). **Xuyang Huang:** Investigation (supporting). **Yong Wang:** Data curation (equal); Funding acquisition (equal); Software (equal); Supervision (equal); Writing‐original draft (equal); Writing‐review & editing (equal).

## Data Availability

The data used to support the findings of this study are available from the corresponding author upon request.
